# Filaggrin Loss-of-Function Mutations Are Risk Factors for Severe Food Allergy in Children with Atopic Dermatitis

**DOI:** 10.3390/jcm10020233

**Published:** 2021-01-11

**Authors:** Annalisa Astolfi, Francesca Cipriani, Daria Messelodi, Matilde De Luca, Valentina Indio, Costanza Di Chiara, Arianna Giannetti, Lorenza Ricci, Iria Neri, Annalisa Patrizi, Giampaolo Ricci, Andrea Pession

**Affiliations:** 1 Department of Translational Medicine, University of Ferrara, 44121 Ferrara, Italy; annalisa.astolfi@unife.it; 2 Pediatric Unit, Department of Medical and Surgical Sciences, S. Orsola-Malpighi Hospital, University of Bologna, 40138 Bologna, Italy; francy.cipriani@gmail.com (F.C.); costanza.dichiara@studio.unibo.it (C.D.C.); arianna.giannetti@libero.it (A.G.); giampaolo.ricci@unibo.it (G.R.); 3 Giorgio Prodi Cancer Research Center, University of Bologna, 40138 Bologna, Italy; matilde.deluca2@studio.unibo.it (M.D.L.); valentina.indio2@unibo.it (V.I.); 4 Department of Experimental, Diagnostic and Specialty Medicine, Division of Dermatology, Azienda Ospedaliero-Universitaria di Bologna Policlinico Sant’Orsola-Malpighi, 40138 Bologna, Italy; lorenza.ricci3@gmail.com (L.R.); iria.neri@aosp.bo.it (I.N.); 5 Dermatology—IRCCS Policlinico di S.Orsola, Department of Experimental, Diagnostic and Speciality Medicine (DIMES), Alma Mater Studiorum University of Bologna, 40138 Bologna, Italy; annalisa.patrizi@unibo.it; 6 Division of Pediatrics, IRCCS Azienda Ospedaliero, Universitaria di Bologna, 40138 Bologna, Italy; andrea.pession@unibo.it

**Keywords:** *Filaggrin*, atopic dermatitis, food allergy

## Abstract

Atopic dermatitis is frequently associated with the onset of other allergic conditions, such as asthma, rhino-conjunctivitis and food allergy. The etiology of atopic dermatitis is marginally understood in spite of the number of predisposing factors, above all, mutations in the *Filaggrin* gene *(FLG*). In this study, the association between loss-of-function variants in the *FLG* gene and other allergic manifestations, in particular food allergy, was evaluated in an Italian pediatric population affected by atopic dermatitis. The 10 more frequently mutated loci in the *FLG* gene were genotyped in 238 children affected by atopic dermatitis and tested for association with clinical features of allergic disorders by a multivariate logistic regression model. R501X and 2282del4 were the only two mutations identified; 12.2% of children carry one of these variants, corresponding to an allelic frequency of 6.5%. According to multivariate statistical analysis, loss-of-function variants in the *FLG* gene represent a risk factor for the onset of severe manifestations of food allergy (OR = 8.9; CI: 3.1–28.3). Peanut and hazelnut were identified as high-risk foods in patients with *FLG* mutations. This study demonstrates that atopic children carrying *FLG* mutations represent a high-risk population due to their predisposition to develop severe food allergy reactions, such as anaphylaxis.

## 1. Introduction

Atopic dermatitis (AD), also known as atopic eczema, is a relapsing inflammatory skin disease with a considerable social and economic burden. It is the most common inflammatory skin disease during childhood with various manifestations over the years and a progression frequently associated with the onset of allergic conditions, especially food allergy (FA), asthma and rhino-conjunctivitis (RC) [1,2].

The complex pathogenesis leading to AD is not yet completely understood, but many hypotheses support the idea of a multifactorial disease involving genetic and immune system alterations along with environmental factors. Since the discovery by Palmer et al. of a strong association between loss-of-function mutations of the *Filaggrin (FLG)* gene and AD and asthma [3], the concept of a skin barrier genetic defect causing AD has replaced the “inside–outside” hypothesis. In the European population, the frequency of the most common *FLG* gene alterations (R501X and 2282del4) varies among different regions, depending on their ethnic group [4,5].

AD and FA are often associated in children: FA may occur in about one third of patients with AD and AD has been associated with earlier and persistent allergic sensitization to foods [6,7]. In children with AD, the early sensitization to food allergens (in particular cow’s milk and hen’s egg) and/or to inhalant allergens represents a risk factor for a more persistent and severe disease and asthma development [8]. Many studies have indicated that transcutaneous allergic sensitization may be facilitated by *FLG* null-mutations, and that both peanut allergy and AD are strongly associated with *FLG* loss-of-function variants [9,10,11,12], as well as other cutaneous conditions [13,14]. In an English population-based birth cohort, the exposure to environmental peanuts early in life (within the first year of life) was associated with peanut sensitization and allergy in children carrying a *FLG* gene mutation [10]. In support of the role of the skin barrier in driving AD, allergic disease and food sensitization, a retrospective follow-up study suggested that the improvement of the skin management in children with AD could reduce or delay the appearance of allergic respiratory diseases [8]. Meanwhile, a recent pilot study with a lipid-rich skin emollient from birth was found to improve skin barrier function and reduce food allergen sensitization [15].

ImmunoglobulinE (IgE)-associated AD is more frequent in childhood than non-IgE-associated AD, and these two phenotypes generally differ in their onset age and remission pattern. Therefore, it is important, especially in early life, to distinguish these two phenotypes in order to evaluate possible allergy-related conditions and to predict the occurrence of other allergic phenotypes later in childhood [16,17].

Considering the wide spectrum of phenotypes possibly associated to *FLG* mutations, this study aimed to identify the association of *FLG* loss-of-function variants with allergic phenotypes, in particular with severe food allergy.

## 2. Experimental Section

### 2.1. Study Population 

Patients suffering from AD evaluated at the Pediatric Allergology and Pediatric Dermatology Outpatient Clinics of the S. Orsola-Malpighi Hospital of Bologna (Bologna, Italy) were recruited for this study. All patients were followed up with clinical examinations at least once a year, or more frequently depending on the severity of their disease and clinical symptoms. The inclusion criteria were: informed consent signed by parents or legal tutors of the child, Italian ancestry, clinical diagnosis of AD within pediatric age (<14 years), formulated on the basis of Hanifin and Rajka criteria [18,19]. Older patients do not imply persistent dermatologic disease, since they were all taken in charge by the Pediatric Allergology Unit.

### 2.2. Ethics

The study was conducted in accordance with the approved guidelines of the Declaration of Helsinki. The study protocol was approved by the Ethical Committee of S.Orsola-Malpighi Hospital of Bologna (code 040/2011/U/Tess). Written informed consent was obtained from all study participants or their legal guardians.

### 2.3. Clinical and Immunologic Evaluation

Clinical data of patients were collected retrospectively, and AD severity was assessed by using the SCORAD index [20]. Atopic sensitization of patients was evaluated by skin prick test (SPT) and by the determination of the total serum IgE and specific IgE. Food allergy was diagnosed through open-label oral food challenge (OFC), following reported guidelines [21], apart from children who had exhibited clinically proven severe food allergy reactions or anaphylaxis episodes.

SPTs (Lofarma, Milan, Italy) were performed for the most common foods (milk and egg) and airborne allergens but also for additional allergens according to the history. The test was considered positive or negative by comparing the allergen wheal with that of histamine. The analysis of sera samples was performed by immune-enzymatic method (ImmunoCAP, ThermoFisher Scientific, Uppsala, Sweden). Total serum IgE levels were defined higher or normal according to the standard values for age, while specific IgE levels were considered positive if higher or equal to 0.35 kU/L for inhalants and 0.70 kU/L for food allergens. Blood samples from patients were collected for the genetic analysis.

### 2.4. FLG Genotyping

**Figure 1:**
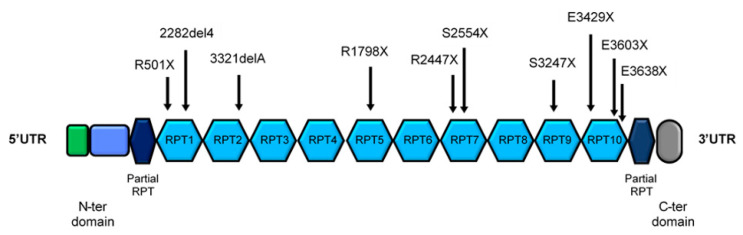
Structure of the coding portion of *FLG* gene. The main part of the protein is encoded by the third and larger exon, having a highly complex and repetitive structure with 10–12 near-identical tandem repeats (RPT). The analyzed loss-of-function variants of the *FLG* gene are reported.

Genomic DNA was extracted from peripheral blood samples using the QIAamp DNA Mini kit (Qiagen, Hilden, Germany). The loci of interest of the *FLG* gene were selected on the basis of the allelic frequency of the loss-of-function mutation reported on the ExAc database [22] and from literature. The analyzed loci were: R501X and 2282del4 (in the first repeat, RPT1), 3321delA (RPT2), R1798X (RPT5), S2554X and R2447X (RPT7), S3247X (RPT9), E3429X, E3603X and R3638X (RPT10) (Figure 1). Target regions were amplified by PCR by means of the AmpliTaq Gold (Thermo Fisher, Waltham, MA, USA). Primers were designed with the Primer Express software v 3.0 (Thermo Fisher, Waltham, MA, USA) in the regions with the highest number of mismatches among the different RPTs of the third exon of the gene and the specificity was verified using Primer Blast [23]. The amplified *FLG* fragments were sequenced by Sanger sequencing procedure with the BigDye Terminator v1.1 Cycle Sequencing kit (Thermo Fisher, Waltham, MA, USA) and loaded on the ABI 3730 DNA Analyzer (Thermo Fisher, Waltham, MA, USA). Primer sequences are available in Supplementary Table S1.

### 2.5. Diagnostic Criteria and Outcome Variables

An indicator variable was built to classify *FLG* mutation carriers and wild-type children, with no distinction between the different kinds of detected mutations since they all produce the premature stop of protein translation. Demographic and clinical data included: sex, presence/absence of an atopic condition in at least one of the patients’ parents, age at AD onset, age at the last clinical evaluation, presence/absence of superinfection at diagnosis time, AD subtype, total IgE serum level, presence/absence of asthma, RC and food allergy.

The presence of a family history of atopy was evaluated considering the diagnosis performed by a physician of at least one of the following conditions: RC, allergic asthma, food allergy, IgE-mediated atopic dermatitis.

Regarding AD, disease severity was defined according to the SCORAD (Scoring Atopic Dermatitis) value, with children with a score <15 classified as affected with a mild form, between 15 and 40 with a medium intensity, and with a score >40 with a severe AD manifestation. Moreover, children with a total serum IgE level ≥100 kU/L or specific IgE value ≥0.35 kU/L were classified as affected with an IgE-associated form.

Concerning food allergy, the presence of FA was clinically evaluated and assessed through open-label oral food challenge (OFC), following reported guidelines [21]. Clinically proven severe food allergy reactions or anaphylaxis episodes were assessed through EAACI (European Academy of Allergy and Clinical Immunology) guidelines, and food-allergic children were stratified into two groups considering symptom severity: mild/moderate and severe FA.22 the last category included children who previously exhibited anaphylaxis episodes and/or required the prescription of an adrenaline auto-injector.

Data on the presence of clinically proven episodes of allergy against specific foods (hazelnut, peanut, egg, cow’s milk, kiwi, fish) and on specific serum IgE level for the tested food allergens (wheat, peanut, hazelnut, albumen, kiwi, milk, cod fish) were also recorded for the food allergy-positive children.

### 2.6. Power Analysis

In Europe, the overall prevalence of clinical symptoms of food allergy confirmed by the detection of positive specific IgE is 3.6% among children [24]. Sample dimension was estimated assuming a frequency of the predisposing allele of 0.12, a food allergy prevalence in childhood of 3%, an allele relative risk = 3 and a type I error rate of 0.05. Under these conditions, we obtained an 80% power to detect an association with 27 cases and 211 controls. The estimates were calculated using the Genetic Power Calculator tool [25].

### 2.7. Statistical Analysis

Statistical analysis was performed through the R software (version 3.1.2).

Correlation between *FLG* mutations and categorical variables was assessed through the chi-square test of independence or the Fisher’s exact test (when dealing with expected frequencies <5). Considering continuous variables, the association with the presence/absence of the *FLG* mutation was analyzed using the non-parametric Wilcoxon–Mann–Whitney test. For each variable, a multivariate logistic regression model was estimated, where the stepwise procedure was used for variable selection. *P*-values ≤ 0.05 were considered statistically significant. When multiple comparisons were performed, the risk was expressed as an odds ratio (OR) and Bonferroni or Benjamini and Hochberg methods were adopted in order to correct the *p*-values for multiple tests.

## 3. Results

### 3.1. Population Description

The database consisted of 238 patients affected with AD, 135 males and 103 females, with a M/F ratio of 1.31. At the enrollment time, the population mean age was 8.2 years (SD: 5.1 years), while at the last clinical evaluation, the mean age was 13.2 years (SD: 4.9 years). The average age at AD onset was equal to 11.6 months (SD: 19.1 months). Clinical features of the population are reported in Table 1.

### 3.2. FLG Mutation Frequency

*Filaggrin* sequencing was performed on 10 recurrent loss-of-function mutation sites: R501X, 2282del4, 3321delA, R1798X, S2554X, R2447X, S3247X, E3429X, E3603X and R3638X (Figure 1), including the most common *FLG* variants analyzed in all the European studies. In the examined cohort of patients, as expected from previous studies, loss-of-function variants were found only in the first repeat (RPT) of the *FLG* gene (R501X and 2282del4). The R501X mutation was present in 16 children, all in heterozygosis except from one carrier of a homozygous mutation. The 2282del4 heterozygous variant was identified in 15 individuals. One patient carried both the loss-of-function *FLG* variants in the RPT1 (Table 2). As a consequence, the total number of *FLG* mutation carriers was 30, representing 12.2% of the studied population. The combined allelic frequency was equal to 6.5% (3.6% for R501X and 3.2% for 2282del4).

### 3.3. FLG Mutation Is not Associated with Asthma and RC

Concerning AD general characteristics, no association with the presence of the *FLG* mutation was detected for sex, age of AD onset, symptoms intensity and family history of atopy (Supplementary Table S2). Since the age of children in the two groups could lead to a selection bias, we confirmed that the age at the last clinical follow-up was not significantly different between the *FLG* mutant and *FLG* wt (12.8 ± 5.3 vs. 13.4 ± 4.9 months; *p*-value = 0.54).

Regarding the distribution of *FLG* variant carriers and AD subtype, 13.4% of the IgE-associated AD patients were carriers of *FLG* variants, while 9.7% of the children with the non-IgE-associated AD form were *FLG* mutated, a difference which is not statistically significant (Supplementary Table S2).

The association between the presence of *FLG* mutation and allergic diseases showed no correlation with asthma and RC. By contrast, FA was found associated with the *FLG* loss-of-function variants (*p*-value= 0.007). Children with the *FLG* variant suffering from FA were 17 (56.6% of the mutated population), seven of them with mild symptoms and 10 (59%) with a severe manifestation. In the wild-type population, children exhibiting allergic reactions to foods were 78 (32.8% of the non-mutated group), 71% with mild symptoms and 29% with a more serious form (Supplementary Table S2).

### 3.4. FLG Mutation Is Associated with Severe FA

In order to identify the variables independently associated with *FLG* mutations, a multivariate logistic regression model was estimated, which showed that the only variables significantly associated with the *FLG* mutation were severe FA and total IgE levels (Table 3 and Table 4).

Only the association between the subgroup of severe food-allergic patients and the presence of *FLG* mutations proved to be significant (OR = 8.94, CI: 3.02–28.27), suggesting that people with a RPT1 *FLG* mutation have a nine-times increased risk of developing severe FA. Conversely, no statistical evidence of an increased risk of developing mild/moderate FA was identified for *FLG*-mutated patients (*p*-value = 0.10). If the analysis was carried out without FA stratification into mild and severe categories, the multivariate analysis showed a smaller but significant OR (3.74, CI: 1.56–9.71), implying that the increased risk observed for the entire FA category was driven by the severe FA subgroup.

Regarding the total IgE variable, there was a small association with *FLG* mutations with a weakly positive OR (1.02, CI: 1.01–1.03), implying that carrying *FLG* mutation has a slightly protective effect on the total IgE level increase among children with DA.

### 3.5. FLG Mutation Carriers Have an Increased Risk of Peanut and Hazelnut Allergy

Since FA seemed to be the most relevant variable associated with the *FLG* mutation, a deeper analysis on specific food sensitization and on allergic reactions was performed in children with and without *FLG* variants. Since specific IgE antibodies are very sensitive to cross-reaction with other kind of allergens, often not belonging to the food category, only the dataset of clinically proven FA patients (*n* = 95) was used for this analysis, thus increasing the confidence that the considered food IgE antibodies were specific for the tested allergens.

**Figure 2:**
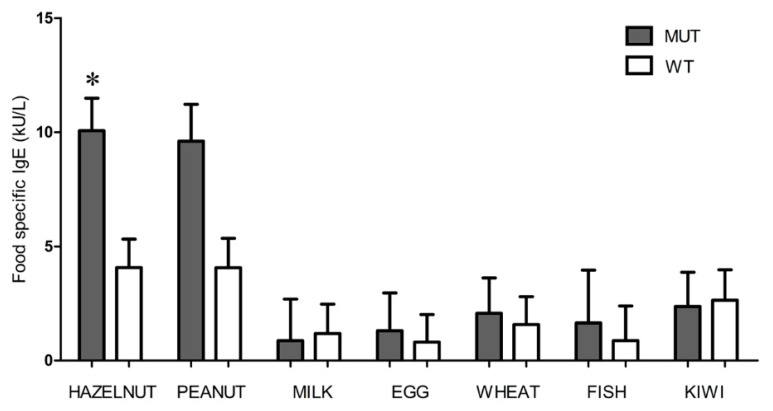
Food specific IgE levels in the *FLG* mutated (grey) and wild-type (white) groups. Geometric IgE mean values with standard error are reported. Hazelnut (*, *p*-value = 0.03) is the only variable with a statistically significant difference between the *FLG* mutated and wild-type groups according to the multivariate model.

The levels of specific IgE antibodies to selected food allergens proved to be higher in the *FLG*-mutated compared to the wild-type population both in the case of peanut and hazelnut (Figure 2), even if the difference reached statistical significance only for hazelnut (*p*-value = 0.03).

The association analysis between the *FLG* genotype and food allergy reactions to specific food allergens was performed again only on clinically proven food-allergic patients (Table 5). The association between each allergen and *FLG* mutations was firstly evaluated using the Chi square test, showing that a significant association was present only in the case of peanut (*p*-value = 0.05) and hazelnut (*p*-value = 0.04). No significant association with *FLG* mutations was found for the other considered food allergens (cow’s milk, egg, fish and kiwi). To prevent multicollinearity, since in the model estimation hazelnut and peanut resulted strongly correlated (*p*-value = 1.7 × 10 − 6, Fisher’s exact test), the regression model was performed once considering all the variables excluding hazelnut and a second time excluding peanut instead of hazelnut. In the first model, only the peanut coefficient emerged as statistically significant (OR = 3.2, CI: 1.1–9.3 and *p*-value = 0.05), while in the second one, a significant association was found for hazelnut (OR = 4.1, CI: 1.5–13.0 and (*p*-value = 0.03), proving that carriers of a RPT1 *FLG* mutation have a four-times increased risk of developing hazelnut allergy and a three-times increased risk of showing peanut allergy.

## 4. Discussion

In the European population, the prevalence of *FLG* gene alterations, especially of R501X and 2282del4 mutations, varies among different regions, depending on the ethnic groups [3,4,5,26,27,28,29,30,31,32].

In our study, 12.2% of patients (30 out of 238) are carriers of *FLG* mutations with an allelic frequency of 6.5% (3.6% for the R501X mutation and 3.2% for 2282del4 variant). This result is in line with the European general tendency even if not coherent with another study performed on an Italian population [28] showing only 1.4% of combined allelic frequency. The heterogeneity in the reported *FLG* mutation frequencies could be explained by the wide spectrum of clinical features of the population under study (i.e., percentage of IgE phenotypes, different grade of severity), thus resulting in a scattered mutation distribution. Moreover, the heterogeneity of inclusion criteria and the type of sequencing approach may influence the percentage of *FLG* mutation carriers.

Previously published papers [3,4,27] showed that *FLG* variants represent a risk factor for asthma and RC development. According to our data, asthma and RC are not associated with *FLG* variants; however, we cannot exclude that in the coming years, given the average age of these patients (13.2 years), some of them will develop asthma and that a significant relationship with asthma occurrence can be uncovered. On the contrary, we can indeed exclude AD as a possible confounding factor, since all the patients included in this study are affected by AD.

The most interesting result of this study is the strong association between severe FA and *FLG* variants. We show that children carrying the R501X or 2282del4 mutations suffer from severe FA almost nine times more than children with wild-type *FLG*. Several reports have already described a correlation between *FLG* variants and FA [10,33,34,35,36], without considering the severity of the allergic reaction. Interestingly, *FLG* RPT1 mutations were identified as predisposing to food allergy also in a genome-wide association study [37], with an OR similar to the one identified in our study when correlating with FA as a whole, and not specifically with severe FA. Moreover, a recent study confirmed the correlation between low *FLG* expression, trans-epidermal water loss and FA in AD children, confirming the same percentage of *FLG*-mutation carriers in AD children with FA (24% vs. 18% in our series) [38]. More importantly, this study confirmed the role of skin barrier dysfunction and food allergy, while our study, beyond confirming association with FA identified in previous studies, shows as a new finding the correlation of *FLG* mutations with disease severity. Indeed, this is the first work analyzing, in a pediatric population with a predominant IgE-associated phenotype, the link between *FLG* mutations and FA focusing on the intensity of the allergic conditions, that shows that *FLG* mutation carriers are a high-risk population for severe allergic reactions as anaphylaxis.

In our study, foods identified as causative of allergic reactions associated with *FLG* variants are peanut and hazelnut (also confirmed by specific IgE sensitization). Other epidemiological and clinical studies [39,40,41,42] reported the connection between *FLG* mutation and peanut allergy, while the hazelnut appeared for the first time in this study. Notably, hazelnut and peanut are highly allergenic foods and they are very common allergens among dried fruit in Italy, so it is reasonable that the severity of FA could be at least partly related to their effect and frequency of use.

The linkage between allergic sensitization and *FLG* mutations is supposed to be represented by the skin barrier defect. An inadequate protection against the external environment allows the contact of environmental allergens, such as food proteins, with the immune system via antigen-presenting cells in the superficial epidermis, leading to sensitization, which could worsen AD symptoms and may also be a precursor condition of food allergies. In these terms, an early identification of the *FLG* mutations in AD children could be important to direct primary and secondary prevention efforts to these allergy-prone children. Taking into account that the onset age of AD does not seem to be associated with the presence of *FLG* mutations, the genetic identification of *FLG*-mutated children could facilitate the implementation of efforts aimed primarily at improving skin barrier function in a high-risk subset of AD children [43]. Nevertheless, the study also shows some limitations, particularly related to the reduced sample size that could limit the power to detect some associations, and to the small number of patients with severe AD included in the cohort. Anyway, these results strongly suggest that the association between severe food allergy and *FLG* mutations warrants further investigations.

In conclusion, this study demonstrated that in the Italian population there is a considerable percentage of children affected by AD carrying an *FLG* mutation (12.2%) and that these loss-of-function mutations are a relevant risk factor for severe FA manifestations. The knowledge of the *FLG* genotype of children with AD could be useful in order to properly treat the disease and all its related clinical manifestations and provide the best preventive measures for this high-risk population.

## Figures and Tables

**Table 1:** Clinical features of the analyzed population (*n* = 238). Data are expressed as number of patients and percentage. The total IgE level variable is reported as the geometric mean with 95% CI.

Variable	Patients *n* (%)
**AD severity**	
Mild	78 (32.8%)
Moderate	127 (53.4%)
Severe	33 (13.8%)
Lesion superinfection	24 (10.1%)
Familiar history of atopy	147 (61.8%)
IgE-associated AD form	201 (86.6%)
Total IgE level	253.1 kU/L (CI: 206.4–310.6 kU/L)
Asthma	80 (33.6%)
RC	136 (57.1%)
**Food allergy**	
Mild/moderate	62 (26.1%)
Severe	33 (13.9%)

**Table 2:** Genotype of the RPT1 *FLG* loci in the 238 patients of the study. One patient was a carrier of both identified RPT1 *FLG* variants in heterozygosis.

	R501X	2282del4	Combined Genotype
Homozygous	1	0	1
Heterozygous	15	15	29
Wild type	222	223	208

**Table 3:** Multivariate model results (*n* = 228). Multivariate model including all the considered covariates obtained from the first step of the selection model procedure.

**Discrete Variable**		***FLG* Mutant (%)** ***n* = 28**	***FLG* wt (%)** ***n* = 200**	**Standard Error**	***p*-Value**	**q-Value** **BH**	**q-Value** **Bonferroni**
Sex	Male	12 (42.9%)	118 (59.0%)	0.45	0.44	0.56	1
AD severity	Mild	7 (25.0%)	65 (32.5%)	Reference category			
	Moderate	17 (60.7%)	106 (53.0%)	0.55	0.23	0.40	1
	Severe	4 (14.3%)	29 (14.5%)	0.75	0.47	0.56	1
Familiar history of atopy *	Yes	8 (28.6%)	68 (34.0%)	0.51	0.54	0.44	1
Asthma	Yes	9 (32.1%)	69 (34.5%)	1.02	0.22	0.40	1
RC	Yes	16 (57.1%)	116 (58.0%)	0.52	0.15	0.37	1
Food allergy	Severe	10 (35.7%)	23 (11.5%)	0.65	**0.00003**	**0.001**	**0.001**
	Mild/moderate	7 (25.0%)	52 (26%)	0.59	0.07	0.22	0.88
**Continuous Variable**		**Mean in *FLG*** **Mutant**	**Mean in *FLG* wt**	**Standard Error**	***p*-Value**	**q-Value** **BH**	**Q-Value** **Bonferroni**
Age of AD onset		7.7 months	12.1 months	0.20	0.63	0.64	1.00
Total IgE level		204.5 kU/L	256.1 kU/L	0.01	**0.001**	**0.003**	**0.01**

Significant values are reported in bold. BH = Benjamini-Hochberg. * Some missing values.

**Table 4:** Multivariate model results, final model (*n* = 228). Only the significant variables emerging from the previous step of the model were considered. “Severe FA” and “Total IgE” are the only variables associated with *FLG* mutations.

**Discrete Variable**		***FLG* Mutant** **(*n* = 28)**	***FLG* wt** **(*n* = 200)**	**OR**	**Standard Error**	***p*-Value**	**q-Value BH**	**q-Value** **Bonferroni**
Food allergy	Severe	10	23	**8.94**	0.56	**0.0001**	0.002	0.0004
	Mild/moderate	7	52	2.55	0.57	0.10	0.10	0.40
**Continuous Variable**		**Mean in *FLG* Mutant**	**Mean in *FLG* wt**	**OR**	**Standard Error**	***p*-Value**	**q-Value** **BH**	**q-Value** **Bonferroni**
Total IgE level		204.54 kU/L	256.1 kU/L	**1.02**	0.006	**0.001**	0.001	0.004

Significant values are reported in bold. BH = Benjamini-Hochberg.

**Table 5:** Association between *FLG* mutations and allergic reactions to different foods. Number of patients, OR, 95% CI and *p*-value of the univariate and multivariate analysis in the subgroup of *FLG*-mutated and wild-type individuals. *n* = 95 (for all the variables there are some missing values).

				Univariate			Multivariate		
Allergen		FLG Mutated (%) n = 17	FLG wt (%) n = 78	OR	95% CI	*P*-Value	OR	95% CI	*p*-Value
Peanut	Yes	11 (64.7%)	26 (33.3%)	3.48	1.09–13.47	**0.05**	**3.2**	1.1–9.3	**0.05**
	No	6 (35.3%)	45 (57.7%)	Reference category					
Hazelnut	Yes	12 (70.6%)	31 (39.7%)	3.17	1.08–10.17	**0.03**	**4.1**	1.5–13.0	**0.03**
	No	4 (23.5%)	36 (46.2%)	Reference category					
Milk	Yes	4 (23.5%)	16 (20.5%)	1.13	0.29–3.73	0.84	Not included in the final model		
	No	13 (76.5%)	59 (75.6%)	Reference category					
Egg (albumen)	Yes	7 (41.2%)	19 (24.4%)	2.06	0.67–6.16	0.20	3.1	0.9–11.0	0.30
	No	10 (58.8%)	56 (71.8%)	Reference category					
Fish	Yes	5 (29.4%)	10 (12.8%)	2.64	0.71–9.08	0.13	3.8	0.9–16.1	0.27
	No	11 (64.7%)	58 (74.4%)	Reference category					
Kiwi	Yes	6 (35.3%)	17 (21.8%)	1.89	0.58–5.77	0.27	Not included in the final model		
	No	11(64.7%)	59 (75.6%)	Reference category					

Significant values are reported in bold.

## Data Availability

The data presented in this study are available on request from the corresponding author. The data are not publicly available due to privacy issues.

## Supplementary Materials

The following are available online at https://www.mdpi.com/2077-0383/10/2/233/s1. Table S1: List of PCR primers; Table S2: Univariate analysis results.
