# Supplementary material for: Filaggrin Loss-of-Function Mutations Are Risk Factors for Severe Food Allergy in Children with Atopic Dermatitis
Source: J Clin Med. 2021 Jan 11;10(2):233. doi: 10.3390/jcm10020233 (PMC7827548; doi:10.3390/jcm10020233)
Supplement: Supplementary file 1 [file jcm-10-00233-s001.pdf]

**Supplementary material**

**Supplementary Table 1. List of PCR primers.**

| Region | Mutation | Primers | Annealing temperature (°C) |
|---|---|---|---|
| RPT1 | R501X<br>2282del4 | F: 5' - AAAGGCTGGGCTGAGACAG - 3'<br>R: 5'-TGGCTCTGCTGATGGTGAC-3' | 62 |
| RPT2 | 3321delA | F: 5' - TCAGGCCATGGACAGGATG - 3'<br>R: 5' - GACACAGCCTGTCCATGAG - 3' | 62 |
| RPT5 | R1798X | F: 5' - GCAGACAGCTCCACAGAC - 3'<br>R: 5' - TTACGTGTTTGTCTGCTGAC - 3' | 60 |
| RPT7 | S2554X<br>R2447X | F: 5' - AAGCAGAAAAACATATGACAAG - 3′<br>R: 5' - AAACTGGATCCCCAGTTCC - 3′ | 60 |
| RPT9-10 | S3247X<br>E3429X | F: 5' - AGCTCCAGGCACTCAGTGTCA - 3'<br>R: 5' - TCATGGTGATGCGACCATGAG - 3' | 64 |
| RPT10 | E3603X<br>R3638X | F: 5' - AGCTCCAGGCACTCAGTG - 3'<br>R: 5' - ATGGTGATGCGACCATGAG - 3' | 60 |

**Supplementary Table 2. Univariate analysis results (N=238).**

P-values refer to the univariate analysis results. In bold are reported the significant values.

* Some missing values.

| **Discrete Variable** | | ***FLG* mutant (%) N=30** | ***FLG* wt (%) N=208** | **P-value** |
|---|---|---|---|---|
| Sex | Male | 12 (40.0%) | 123 (59.1%) | 0.05 |
| Symptoms intesity | Mild | 9 (30.0%) | 69 (33.2%) | Reference category |
| | Moderate | 17 (56.7%) | 110 (52.9%) | 0.70 |
| | Severe | 4 (13.3%) | 29 (13.9%) | 0.93 |
| Familiar history of atopy* | Yes | 19 (63.3%) | 128 (61.5%) | 0.74 |
| AD subtype* | IgE-associated | 27 (90.0%) | 174 (83.7%) | 0.52 |
| Asthma | Yes | 10 (33.3%) | 70 (33.7%) | 0.97 |
| RC | Yes | 17 (56.7%) | 119 (57.2%) | 0.96 |
| Food allergy | Mild/moderate | 7 (23.3%) | 55 (26.4%) | 0.63 |
| | Severe | 10 (33.3%) | 23 (11.1%) | **0.007** |
| **Continuous Variable** | | **Mean in *FLG* mutant (± SD or CI)** | **Mean in *FLG* wt (± SD or CI)** | **P-value** |
| Age of AD onset | | 8.1 months (±12.2) | 12.1 months (±19.8) | 0.29 |
| Total IgE level | | 204.6 kU/L (98.2-425.9) | 260.9 kU/L (211.3-322.0) | 0.72 |
